# Comprehensive Secondary Metabolite Profiling Toward Delineating the Solid and Submerged-State Fermentation of *Aspergillus oryzae* KCCM 12698

**DOI:** 10.3389/fmicb.2018.01076

**Published:** 2018-05-25

**Authors:** Su Y. Son, Sunmin Lee, Digar Singh, Na-Rae Lee, Dong-Yup Lee, Choong H. Lee

**Affiliations:** ^1^Department of Bioscience and Biotechnology, Konkuk University, Seoul, South Korea; ^2^NUS Synthetic Biology for Clinical and Technological Innovation (SynCTI), Life Sciences Institute, National University of Singapore, Singapore, Singapore; ^3^Bioprocessing Technology Institute, Agency for Science, Technology and Research (A^*^STAR), Singapore, Singapore; ^4^School of Chemical Engineering, Sungkyunkwan University, Suwon, South Korea

**Keywords:** *Aspergillus oryzae*, solid state fermentation, submerged fermentation, metabolomics/metabolite profiling, antimicrobial activity

## Abstract

*Aspergillus oryzae* has been commonly used to make *koji, meju*, and soy sauce in traditional food fermentation industries. However, the metabolic behaviors of *A. oryzae* during fermentation in various culture environments are largely uncharacterized. Thus, we performed time resolved (0, 4, 8, 12, 16 day) secondary metabolite profiling for *A. oryzae* KCCM 12698 cultivated on malt extract agar and broth (MEA and MEB) under solid-state fermentation (SSF) and submerged fermentation (SmF) conditions using the ultrahigh performance liquid chromatography-linear trap quadrupole-ion trap-mass spectrometry (UHPLC-LTQ-IT-MS/MS) followed by multivariate analyses. We observed the relatively higher proportions of coumarins and oxylipins in SSF, whereas the terpenoids were abundant in SmF. Moreover, we investigated the antimicrobial efficacy of metabolites that were extracted from SSF and SmF. The SSF extracts showed higher antimicrobial activities as compared to SmF, with higher production rates of bioactive secondary metabolites *viz*., ketone-citreoisocoumarin, pentahydroxy-anthraquinone, hexylitaconic acid, oxylipins, and saturated fatty acids. The current study provides the underpinnings of a metabolomic framework regarding the growth and bioactive compound production for *A. oryzae* under the primarily employed industrial cultivation states. Furthermore, the study holds the potentials for rapid screening and MS-characterization of metabolites helpful in determining the consumer safety implications of fermented foods involving *Koji* mold.

## Introduction

*Aspergillus* species are a ubiquitous mold, which widely affects human life in both deleterious and beneficial ways, since some species are pathogens and the others are used to make fermented foods (Kang et al., 2013). Traditionally, *A. oryzae* (*koji* mold) has been used to produce a variety of fermented foods and beverages, such as *deonjang, sake, makgeoli*, and *miso* (Lee et al., 2017). The fermented foods are made using spontaneously occurring or inoculated microbial species with selective microflora, including mold (*Aspergillus*), yeast, and bacterial species to colonize and modulate the food substrates (Chilton et al., 2015). Recently, metabolomics studies of fermented food under different culture conditions have highlighted distinct profiles of metabolites owing to differences in microbial consortia and metabolism (Oh et al., 2016; Lee et al., 2017). Furthermore, the dynamics of microbial communities vary spatiotemporally in a variety of fermentative systems, which have been investigated to determine the quality of end-products (Chen et al., 2014; Yang et al., 2016b). Specifically, these studies have suggested that *A. oryzae* is among the dominant mold varieties in both the natural as well as the *in situ* cultivated microenvironments i.e., fermented foods.

Previous studies have established that the physicochemical parameters (pH, aeration, and incubation temperature) and nutrients (carbon and nitrogen sources) categorically affect mold growth and metabolism (Pansuriya and Singhal, 2010). Typically, the microorganisms detect environmental changes and adapt themselves to survive in altered conditions through maneuvering their metabolism, especially the secondary metabolite production (Arumugam et al., 2014). The secondary metabolites syntheses facilitate microbial growth and survival through acting as pathogenicity determinants or extracellular signaling molecules among population of competing microbial communities (Arumugam et al., 2014). In particular, fungal secondary metabolites are imperative compounds for antimicrobial substances, antibiotics, alkaloids, toxins, and pigments syntheses with promising applications in food and pharmaceutical industries (Arumugam et al., 2014). To increase the production of secondary metabolites, many studies have considered optimizing the culture conditions, such as solid-state fermentation (SSF) and submerged fermentation (SmF) (Miranda et al., 2014; Hansen et al., 2015). Previously, we have highlighted the subtle effects of SSF and SmF on the distinct metabolomes and transcriptomes in *Penicillium expansum* (Kim et al., 2016).

Metabolomics involve unbiased analysis of the overall complements of metabolites in a biological sample under a given set of conditions, serving as a technique to provide the important information of cell, tissue, or whole organism physiologies (Singh et al., 2017). The mass spectrometry (MS)-based metabolomic profiles coupled with multivariate statistical analysis help in determining the effects of different culture conditions as a function of metabolomic and transcriptomic disparity in molds (Kim et al., 2016). Preparative HPLC (prep-HPLC) is the useful tool for the isolation and purification of metabolites such as flavonoid, sterols, and ester, and offers various advantages *viz*., high method reproducibility and robustness as well as efficient compound recovery useful for screening bioactive fractions (Lee et al., 2013; Dang et al., 2016). A holistic assessment of the metabolomes influenced by two different fermentation states i.e., SSF and SmF, of *A. oryzae* offers insights into their global and non-targeted metabolic changes. Furthermore, the approach may potentially delineate the chemotaxonomic characterization of koji mold under the two important culture conditions.

In this study, we report the effects of different culture conditions i.e., SSF and SmF, on untargeted secondary metabolite profiles of *A. oryzae* KCCM 12698. The metabolite profiles were evaluated and tentatively identified using the ultrahigh performance liquid chromatography-linear trap quadrupole-ion trap-mass spectrometry (UHPLC-LTQ-IT-MS/MS) datasets. The study aims to delineate the metabolomic disparity between the two primary cultivation states i.e., SSF and SmF, for *A. oryzae*. Herein, we highlighted the metabolic disparity between the two primary cultivation states of *Aspergillus oryzae* (trivially: *Koji* mold) employed in various fermented food (*deonjang, sake, makgeoli*, and *miso*) as well as industrial production of value added products (pharmacologically active, cosmetic, or food additive compounds). Hence, the comprehensive profiling of the secondary metabolites might illuminate the range of secreted metabolic classes helpful in delineating the optimal fermentative conditions and associated implication of secreted end products, either beneficial or detrimental.

## Materials and methods

An illustrative outline of experimental procedure is presented in Supplementary Figure 1.

### Chemicals and reagents

Acetonitrile, ethyl acetate, methanol, and water were purchased from Fisher Scientific (Pittsburgh, PA, USA). Malt extract, yeast extract, tryptic soy agar (TSA), nutrition agar (NA), and peptone were purchased from Becton Dickson (Franklin Lakes, NJ, USA). Agar powder and glucose were purchased from Junsei Chemical (Tokyo, Japan). Metabolite standard 12, 13-DiHOME was purchased from Cayman Chemical (Ann Arbor, MI). Antibiotics ampicillin, amphotericin B were purchased from Sigma-Aldrich (St. Louis, MO, USA).

### Strains and media

*A. oryzae* KCCM 12698 were obtained from the Korean Culture Center of Microorganisms (KCCM, Republic of Korea). *A. oryzae* was maintained on malt extract agar (MEA; 20 g malt extract, glucose: 20 g, 1 g peptone and 20 g agar per liter) at 28°C.

For the antimicrobial activity, *Candida albicans* KACC 30062, *Escherichia coli* KCTC 1682, *Staphylococcus aureus* KACC 1621 were used. These strains were obtained from the Korean Agricultural Culture Collection (KACC, Korea) and the Korean Collection for Type Cultures (KCTC, Korea). Tested microbials were maintained on different media. *C. albicans* was cultured on yeast malt agar (YMA; 3 g yeast extract, 3 g malt extract, 5 g peptone, 10 g glucose, and 20 g agar per liter) at 28°C. *E. coli* and *S. aureus* were cultured on nutrition agar (NA; 23g nutrition agar per liter) at 37°C.

### Preparation of spore suspension

*A. oryzae* was transferred from frozen stock (−80°C) to MEA plates for pre-culture incubation, prior to the harvesting of viable spores. A 12-day old culture of the strain was treated with 0.01% Tween-20. Spores were collected by washing the surface of the strain and counted using Neubauer chamber. The final spore inoculum size was adjusted to approximately 3.0 × 10^6^ spores/mL.

### Culture condition

For SSF, 100 mL of MEA plates were inoculated with 1 mL of spore suspension (3.0 × 10^6^ spores/mL) into 150 × 25 mm petri dishes and maintained at 28°C. For SmF, 100 mL of malt extract broth (MEB) medium in 250 mL Erlenmeyer flasks was inoculated with 1 mL of spore suspension (3.0 × 10^6^ spores/mL) and incubated at 28°C and 200 rpm. The *A. oryzae* culture in the two different fermentation states (SSF and SmF) were harvested at regular intervals 0 day to 16 days at every 4 days interval, and the samples immediately stored at deep freezing conditions (−80°C) until analyses (Supplementary Figure 2). Three biological replicates were collected each for SSF and SmF conditions representing each time interval.

### Extraction of fungal metabolites

Extraction of Fungal metabolites was followed using the method partially adopted from Kim et al. (2016). The metabolites from SSF cultured *A. oryzae* were extracted by adding 100 mL of ethyl acetate to the chopped agar plugs followed by agitation under a shaking incubator at 200 rpm for 24 h. On the other hand, the metabolites from SmF culture were extracted using solvent-partitioning method with ethyl acetate (1:1, v/v) under similar conditions (200 rpm) for 24 h. The samples were centrifuged (5,000 × g) for 10 min at 4°C (Universal 320 R, Hettich, Zentrifugen, Germany). The partially purified solvent extracts were dried using a speed vacuum concentrator and re-suspended in MeOH at appropriate dilutions. The solvents extracts were filtered using 0.45 μm disposable polytetrafluoroethylene (PTFE) filter prior to LC-MS analysis. The final concentration of each sample was adjusted to 20 mg/mL.

### UHPLC-LTQ-IT-MS/MS and UPLC-Q-TOF-MS analysis

UHPLC-LTQ-IT-MS/MS analysis was performed using the method partially adapted from Oh et al. (2016) on the LTQ-XL ion trap mass spectrometer equipped with an electrospray interface (Thermo Fisher Scientific, San José, CA) coupled with DIONEX UltiMate 3000 RS Pump, RS Autosampler, RS Column Compartment and RS Diode Array Detector (Dionex Corporation, Sunnyvale, USA). The samples were separated on a Thermo Scientific Syncronis C18 UHPLC column with a 1.7 μm particle size. The mobile phase consisted of 0.1% formic acid in water (solvent A) and 0.1% formic acid in acetonitrile (solvent B), with the gradient flow program as follows: the initial solvent condition was 10% of solvent B; the gradient was then gradually increased from 10% solvent B to 100% solvent B over 18 min. Following this, solvent B was decreased to 10% and maintained as such for next 4 min, completing the total run time of 22 min. The flow rate was maintained at 0.3 mL/min with a 10 μL injection volume. The photodiode array detector was set at a wavelength range of 200–600 nm and was managed by the 3D Field. Mass spectra were recorded using electrospray ionization (ESI) at both positive and negative ion modes spanning a mass range of 150–1,000 m/z. The various operational parameters used were as follows: source voltage, ± 5 kV; capillary voltage, 39 V; and capillary temperature, 275°C. Tandem MS analysis was performed by scan-type turbo data-dependent scanning under the conditions used for negative mode MS scanning. The sample analysis was performed for 3 biological replicates. To decrease the effects of systematic error, the samples were analyzed in random blocks of ten runs followed by an intermitted quality control (QC) sample made with pooled blends from each sample extracts (Godzien et al., 2015).

UPLC-Q-TOF-MS analysis was followed by our previous study (Lee et al., 2017). Validations of selected metabolites were performed with a UPLC-Q-TOF-MS analysis. Waters Micromass QTOF Premier used an UPLC ACQUITY system (Waters, Milford, MA) equipped with a binary solvent delivery apparatus, an auto-sampler, and an ultraviolet (UV) detector. The column selected was an ACQUITY UPLC BEH C18 column (100 mm × 2.1 mm × 1.7 μm particle size, Waters Corp.). The operation parameters were set as follows: injection volume, 5 μL; flow rate, 0.3 mL/min; and column temperature, 37°C. The mobile phase consisted of 0.1% formic acid in water (A) and 0.1% formic acid in acetonitrile (B). The gradient program was set as follows: 5% solvent B was maintained initially for 1 min followed by a gradual increase to 100% over 9 min, and then maintained at 100% B for next 1 min, with a subsequent decrease to 5% over final 3 min, maintaining a total runtime of 14 min. The MS data were collected in the range of 100–1,000 m/z using Waters Q-TOF Premier system (Micromass MS Technologies, Manchester, U.K.) under negative- and positive-ion modes. The capillary voltage and cone voltage were set at 2.5 kV and 50 V, respectively. The source temperature was set at 100°C, with the desolvation gas (nitrogen) and cone gas (nitrogen) flow rates tuned to 600 and 50 L/h, respectively.

### Data processing and statistical analysis

The UHPLC-LTQ-IT-MS/MS data were obtained from Xcalibur software (version 2.00, Thermo Fisher Scientific), and raw data were subsequently converted to netCDF (^*^.cdf) format using Xcalibur software. The MS data files were then processed using MetAlign software (RIKILT-Institute of Food Safety, Wageningen, Netherlands) to estimate the retention times, normalized peak intensities, and accurate masses. The results of alignment data were exported to Excel files (Microsoft, Redmond, WA, USA), and multivariate statistical analyses were performed using SIMCA-P+ software (version 12.0, Umetrics, Umea, Sweden). Principal component analysis (PCA), partial least squares discrimination analysis (PLS-DA), and the loading plots were employed to compare distinctive metabolites between SSF and SmF. The variable importance in the projection (VIP) value and analysis of variance (ANOVA) methods were used for the tentative identification of significantly different metabolites detected from UHPLC-LTQ-IT-MS/MS (at VIP > 0.7, *p* < 0.05) analyses. Statistical analysis for the data involving *t*-test was performed on PASW Statistical software (version 18.0, SPSS, Inc., Chicago, IL, USA). Tentative metabolite identification procedure was followed by Lynn et al. (2015). The selected secondary metabolites were putatively identified based on various information such as mass spectra, MS^n^ fragment, retention time, UV spectrum, elemental composition, mDa, and i-Fit data obtained from UHPLC-LTQ-IT-MS/MS and UPLC-Q-TOF-MS as well as comparing with standard compound, the published references, Dictionary of Natural Product (CCD, Copyright 2008, Taylor & Francis Group, Boca Raton, FL, USA) and Antibase 3.0 (CambridgeSoft Corporation, Cambridge, MA, USA).

### Disk diffusion method for evaluation the antimicrobial activity

The antimicrobial bioactivities for *A. oryzae* SSF and SmF extracts harvested at regular intervals (0, 4, 8, 12, 16 days) against two bacterial (*E. coli* and *S. aureus*) and one fungal (*C. albicans*) species were assayed using the disk diffusion method with few modification (Klančnik et al., 2010; Kang et al., 2013). 100 μL of each microbial suspension was seeded and spread uniformly on NA and YMA plates, respectively. Whatman sterile filter paper disks (6 mm in diameter) were loaded with 10 μL of *A. oryzae* extract dissolved in 100% methanol at appropriate concentration (10 mg/mL). The extract loaded paper disks were placed onto the agar surface with microbial cultures. Antibiotic (ampicillin and amphotericin B) loaded paper disk were used as positive control and 100% methanol which is the same solvent used to dissolve the *Aspergillus* extract served as a negative control. The assay plates were incubated for 1 day at 37°C. For each bioactivity assay, pooled three biological replicates were carried out in triplicate.

### Preparative HPLC for purifying bioactive compounds

Preparative high-performance liquid chromatography (HPLC) was followed as previously described by Lee et al. (2013) with minor modification. The sample separation was performed on a YMC-Pack Pro C18 reversed-phase column (250 × 4.6 mm × 5 μm particle size) with pump L-2130 and L-2455 diode array detector (Hitachi, Tokyo, Japan) using 5% acetonitrile in water (solvent A) and 100% acetonitrile (solvent B) following the gradient: 5% solvent B was maintained initially for 2 min followed by a gradual increase to 100% over 55 min, then equilibrated at 100% solvent B for 2 min, and sharply decreased to 5% over the final 1 min. The flow rate was 1 mL/min and the peaks were monitored at a wavelength of 220 nm. A total of 60 time-based fractions at every 1 min were collected and dried subsequently using speed vacuum concentrator. Before analyzing preparative HPLC, 4 mg of *A. oryzae* extracts was injected for HPLC profiling. The flow rate was 1 mL/min and the photodiode array was set at 220–600 nm. Followed by HPLC profiling, 100 mg of *A. oryzae* extract was injected for preparative HPLC. The flow rate was 1 mL/min and the peaks were monitored at a wavelength of 220 nm (Supplementary Figure 8A). Overall, sixty fractions were collected at the rate of 1 min^−1^, and dried subsequently by speed vacuum concentrator. The fractions were reconstituted in 100% methanol (1 mg/mL) and the corresponding antimicrobial activities were examined using the method described in previous section for three analytical replicates. The bioactive fractions were analyzed by UHPLC-LTQ-IT-MS/MS and UPLC-Q-TOF-MS methods.

## Results

### Non-targeted metabolite profiling of *A. oryzae* in SSF and SmF

**Figure 1:**
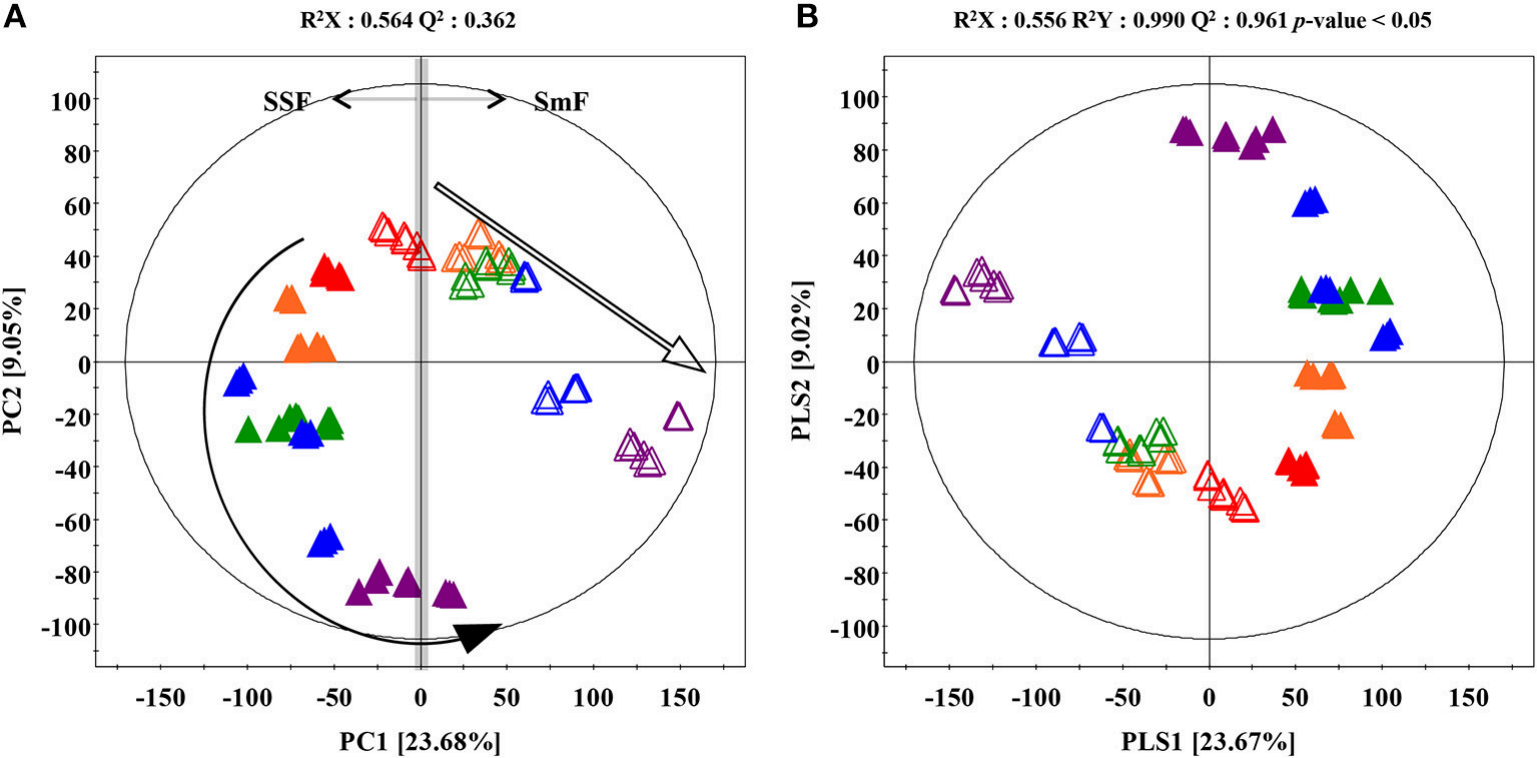
**(A)** Principal component analysis (PCA) and **(B)** partial least squares-discriminant analysis (PLS-DA) score plot derived from the UHPLC-LTQ-IT-MS/MS datasets for *A. oryzae* KCCM 12698 during SSF and SmF conditions. The arrow indicate the flow of times changes. (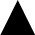, Solid-state fermentation; 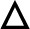, Submerged fermentation; 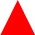, 0 day; 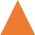, 4 days; 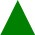, 8 days; 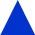, 12 days; 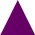, 16 days).

The time correlated (0, 4, 8, 12, 16 days) metabolite profiling datasets such as LC-MS chromatogram from *A. oryzae* extracts exhibited different patterns for SSF and SmF cultivation (Supplementary Figure 3). Multivariate analyses including the unsupervised principle component analysis (PCA), as well as the supervised partial least squares discrimination analysis (PLS-DA) were performed to select the discriminant metabolites adding to the observed variance. The PLS-DA score plots based on UHPLC-LTQ-IT-MS/MS profiling data in negative ion mode showed distinct metabolomic patterns between SSF and SmF along PLS1 (23.68%) and PLS2 (9.02%) components (Figure 1B). The statistical variants for the PLS-DA plots were indicated using R^2^X (0.556) and R^2^Y (0.990), representing the total sum of squares. Whereas, the fraction of total variation for X and Y components was signified by Q^2^ (0.961). A similar pattern for metabolomics data was observed using the PCA score plot (Figure 1A). The significantly discriminant metabolites between SSF and SmF were selected using the variable importance in projection value (VIP > 0.7) and *p*-value (*p* < 0.05). The selected metabolites were putatively identified using their retention time, mass spectra, λ_max_, MS^n^ fragment patterns, and elemental compositions based on the UHPLC-LTQ-IT-MS/MS and UPLC-Q-TOF-MS datasets, as well as the published reports and standard compound and presented all of ion extracted chromatogram, MS spectrum, and UV spectrum to clearly understand metabolite identification (Supplementary Figures 6, 7). A total of 26 significantly discriminant metabolites were putatively identified (VIP > 0.7, *p* < 0.05) and subsequently categorized in to the following secondary metabolite classes, including two coumarins *viz*., dihydroxy-methoxycoumarin (1), ketone-citreoisocoumarin (2); four terpenoids *viz*., asperaculin A (3), austalide F (4), austalide H (5), meroterpenoid derivatives (6); seven unsaturated fatty acids *viz*., 9,12,13-TriHODE (7), 9,12,13-TriHOME (8), 9,10,13-TriHOME (9), 8,11-DiHODE (10), 12,13-DiHODE (11), 12,13-DiHOME (12), 7,10-DiHOME (13); and four miscellaneous metabolites *viz*., diketopiperazine containing dihydroxy-methoxy-phenylalanine (14), pentahydroxy-anthraquinone (15), dihydroxydodecanoic acid (16), and hexylitaconic acid (18) (Table 1).

**Table 1:** Tentative identified metabolites from *A. oryzae* KCCM 12698 according to different fermentation conditions based on UHPLC-LTQ-IT-MS/MS and UPLC-Q-TOF-MS data.

**UHPLC-LTQ-IT-MS/MS**								**UPLC-Q-TOF-MS**				
**No**.	**Rt^a^ (min)**	**Tentative identification^b^**	**Measured mass (m/z)**		**M.W.^c^**	**MS^n^ mass fragment**	λ _max_	***m/z***	**Molecular formula**	**mDa**	**i-Fit**	**ID^d^**
			**[M+H]^+^**	**[M–H]^−^**				**[M–H]^−^**		**(Error)**	**(Norm)**	
**COUMARIN**												
1	7.06	Dihydroxy-methoxycoumarin	209	207	208	209> 179> 163	279, 366, 382(sh)	207.0280	C10H7O5	−1.1	0.662	Antibase
2	8.66	Ketone-citreoisocoumarin	279	277	278	277> 233, 219, 191	206, 277, 366, 381	277.0713	C14H13O6	0.1	0.798	Ref(CCD)
**TERPENOID**												
3	9.21	Asperaculin A	281	279	280	279> 235> 217	210	279.1216	C15H19O5	−0.4	0.251	Ingavat et al.
4	13.58	Austalide F	981^e^	489	490	489> 471, 445, 223, 205	220	489.2171	C26H33O9	1.5	2.346	Ref(CCD)
5	14.23	Austalide H	–	521^f^	476	521> 475, 457, 431, 221	222	475.2340	C26H35O8	1.2	1.290	Ref(CCD)
6	15.55	Meroterpenoid derivatives	489	487	488	487> 443> 383, 357, 339	223	487.2328	C28H39O7	0.4	1.131	Matsuda et al., 2013
**UNSATURATED FATTY ACID**												
7	10.05	9,12,13-TriHODE	329	327	328	327> 309, 291, 229, 211, 183	211, 280	327.2163	C18H31O5	−0.5	1.425	Nørskov et al., 2013
8	10.68	9,12,13-TriHOME	311	329	330	329> 311, 293, 229, 211	277(sh)	329.2332	C18H33O5	0.0	0.112	Strassburg et al., 2012
9	11.26	9,10,13-TriHOME	331	329	330	329> 311, 293, 171, 139	201	329.2332	C18H33O5	0.0	0.112	Strassburg et al., 2012
10	12.08	8,11-DiHODE	313	311	312	311> 293> 195, 157	221, 277(sh)	311.2280	C18H31O4	−0.1	0.896	Shin et al., 2016
11	12.74	12,13-DiHODE	625^e^	311	312	311> 293, 183	219, 274(sh)	311.2222	C18H31O4	−0.1	0.896	Strassburg et al., 2012
12	13.41	12,13-DiHOME	315	313	314	313> 295, 183	205	313.1571	C18H33O4	−1.1	0.027	STD
13	14.41	7,10-DiHOME	315	313	314	313> 295, 269, 171, 157, 141	222	313.1571	C18H33O4	−1.1	0.027	Nilsson et al., 2010
**MISCELLANEOUS**												
14	7.22	Diketopiperazine containing dihydroxy-methoxyphenylalanine	408	452	407	452> 406> 391> 261> 176	200, 276, 366(sh)	406.1375	C22H22N3O5	2.2	1.145	Lin et al., 2008
15	9.85	Pentahydroxy-anthraquinone	289	287	288	287> 259, 243, 231	214, 279, 366, 405	287.0164	C14H7O7	−4.5	0.003	Ref(CCD)
16	9.89	Dihydroxydodecanoic acid	247	245	246	245> 227, 199, 185	214	245.1738	C13H25O4	−1.7	0.055	Ref(CCD)
17	10.05	Glycerol-hydroxydodecanoic acid^g^	291	289	290	–	214, 275(sh)	289.2009	C15H29O5	−0.6	0.261	Ref(CCD)
18	11.75	Hexylitaconic acid	215	213	214	213> 169	213	213.1127	C11H17O4	0.7	0.537	Ref(CCD)
**UNKNOWN**												
19	8.98	N.I. 1	441	439	440	439> 407> 361, 317> 273	210	439.1595	C21H27O10	0.0	0.297	–
20	9.04	N.I. 2	457	455	456	455> 227> 141	205, 366	455.1913	C22H31O10	−0.3	1.430	–
21	9.54	N.I. 3	397	395	396	395> 349, 305, 261	213	395.1691	C20H27O8	−1.5	0.256	–
22	10.36	N.I. 4	465 ^e^	231	232	231> 213, 169	202, 287, 366	231.1231	C11H19O5	−0.6	0.481	–
23	11.70	N.I. 5	339	337	338	337> 309, 293	217, 366, 381	337.0345	C18H9O7	−0.9	3.508	–
24	14.78	N.I. 6	587	585	586	585> 411, 191	219	585.2553	C28H41O13	0.9	0.350	–
25	15.32	N.I. 7	563	561	562	561> 387, 191, 173	220	561.4375	C31H61O8	0.7	1.231	–
26	15.72	N.I. 8	–	413	–	413> 395, 333, 309	224	413.2898	C23H41O6	0.1	1.159	–
27	15.79	N.I. 9	589	587	588	587> 413, 191	223	587.2680	C28H43O13	3.0	1.286	–

a *Retention time*;

b *Tentative metabolites based on variable importance projection (VIP) > 0.7 and p-value < 0.05*;

c *Molecular weight*;

d *Identification. STD, commercial standard compound; CCD, The dictionary of natural products*;

e *[2M+H]^+^*;

f *[M+FA–H]^−^*;

g *Detected only in prep-HPLC fraction*.

*TriHODE, Trihydroxyoctadecadienoic acid; TriHOME, Trihydroxyoctadecenoic acid; DiHODE, Dihydroxyoctadecadienoic acid; DiHOME, Dihydroxyoctadecenoic acid*.

### Evaluation of the relative levels of significantly discriminant metabolites between SSF and SmF

**Figure 2:**
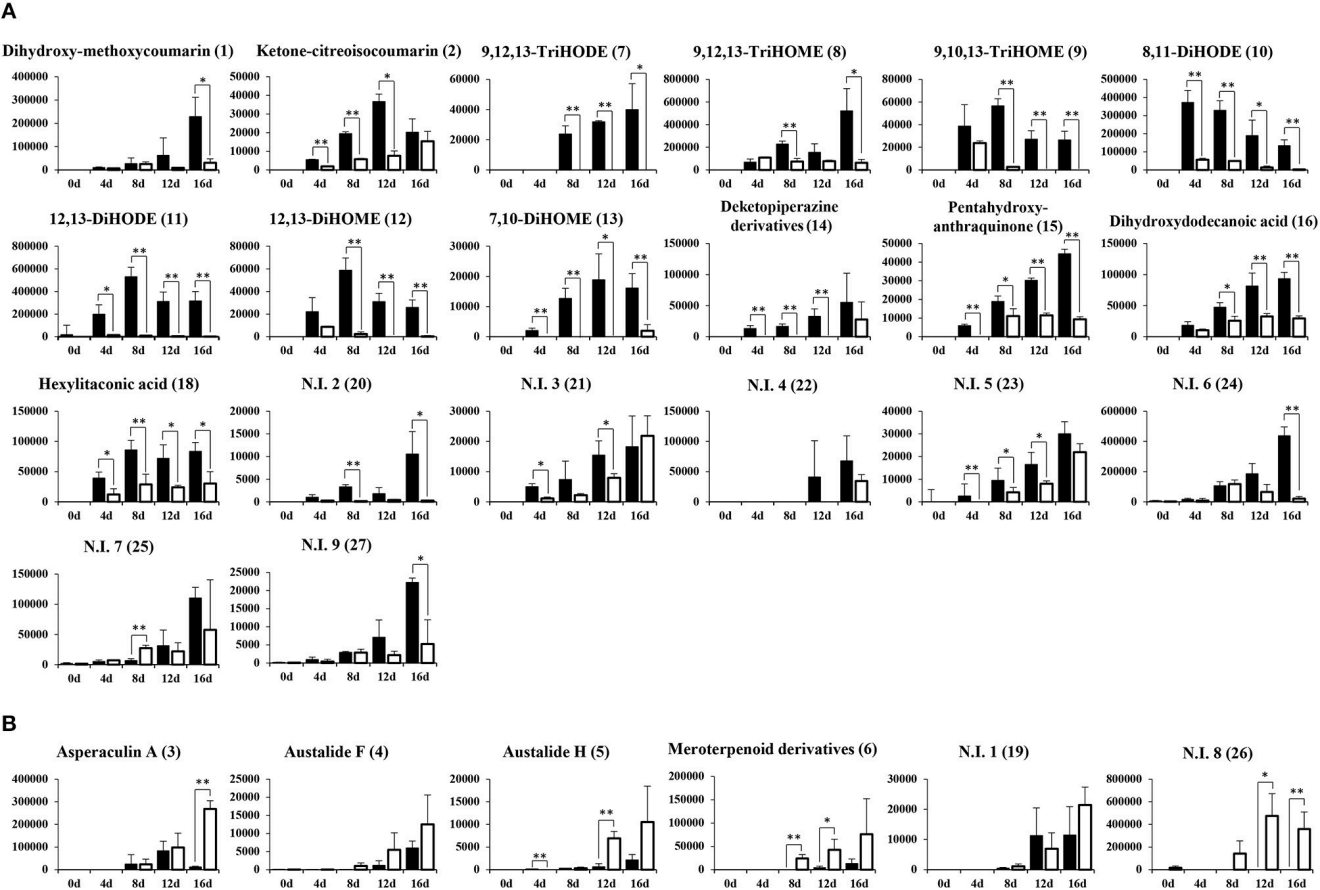
Production of secondary metabolites from solid-state **(A)** and submerged **(B)** fermentation of *A. oryzae* KCCM 12698. The *Y-axis* of the graphs indicates peak area of each metabolite normalized by volume (100 mL). Data are shown as means ± S.D. (*n* = 9). Significant differences between the SSF and SmF groups were identified by *t*-test (^*^p < 0.05, ^**^p < 0.01). (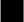, Solid-state fermentation; 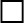, Submerged fermentation).

**Figure 3:**
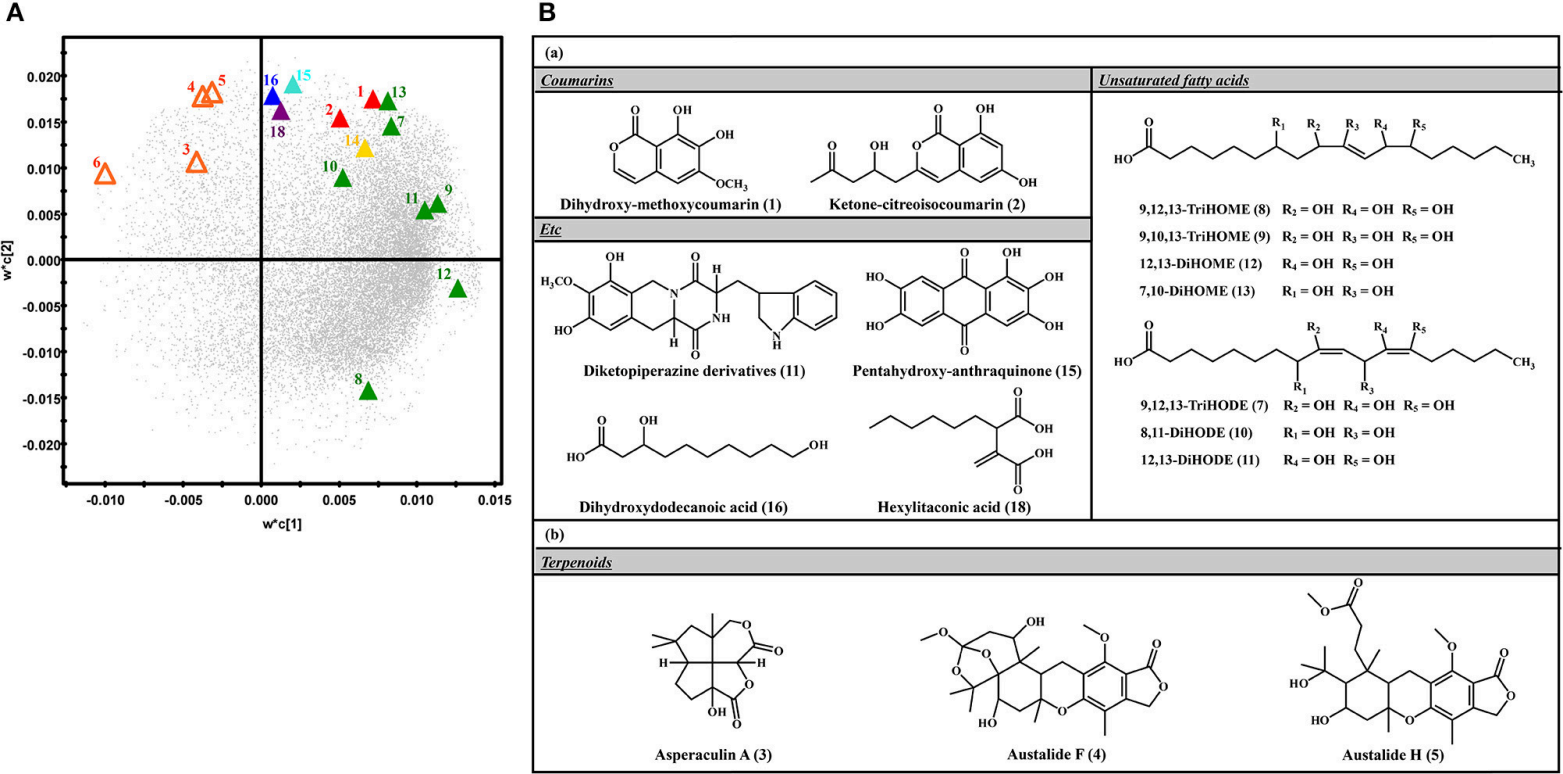
**(A)** PLS-DA loading plots of SSF and SmF samples analyzed using UHPLC-LTQ-IT-MS/MS. 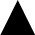, Solid-state fermentation; 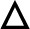, Submerged fermentation; 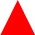, Coumarins; 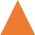, Terpenoids; 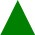, Unsaturated fatty acids; 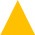, Diketopiperazine; 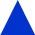, Anthraquinone; 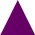, Saturated fatty acid; 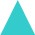, Organic acid. **(B)** The structure of compounds significantly produced in **(a)** solid-state fermentation and **(b)** submerged fermentation.

The relative levels of 26 significantly discriminant secondary metabolites were expressed by the corresponding peak area from UHPLC-LTQ-IT-MS/MS chromatograms (Figure 2). Among 26 selected metabolites, 20 metabolites, including coumarins (1, 2), unsaturated fatty acids (7–13), diketopiperazine derivatives (14), pentahydroxy-anthraquinone (15), dihydroxydodecanoic acid (16), and hexylitaconic acid (18), were relatively more abundant in SSF extracts. The remaining six metabolites, including terpenoids (3–6) and two N.I metabolites (N.I. 1 and 9), were comparatively higher in SmF extracts. Further, the loading plots derived from PLS-DA datasets coupled with sub-group of chemical structure for discriminative metabolites and their relative contents showed that coumarins (1–2), unsaturated fatty acids (7–13), diketopiperazine derivatives (11), pentahydroxy-anthraquinone (15), dihydroxydodecanoic acid (16), and hexylitaconic acid (18) were observed mainly in SSF, whereas terpenoids (3–5) were primarily abundant in SmF (Figure 3).

### Evaluation and partial purification of antimicrobial metabolites

**Figure 4:**
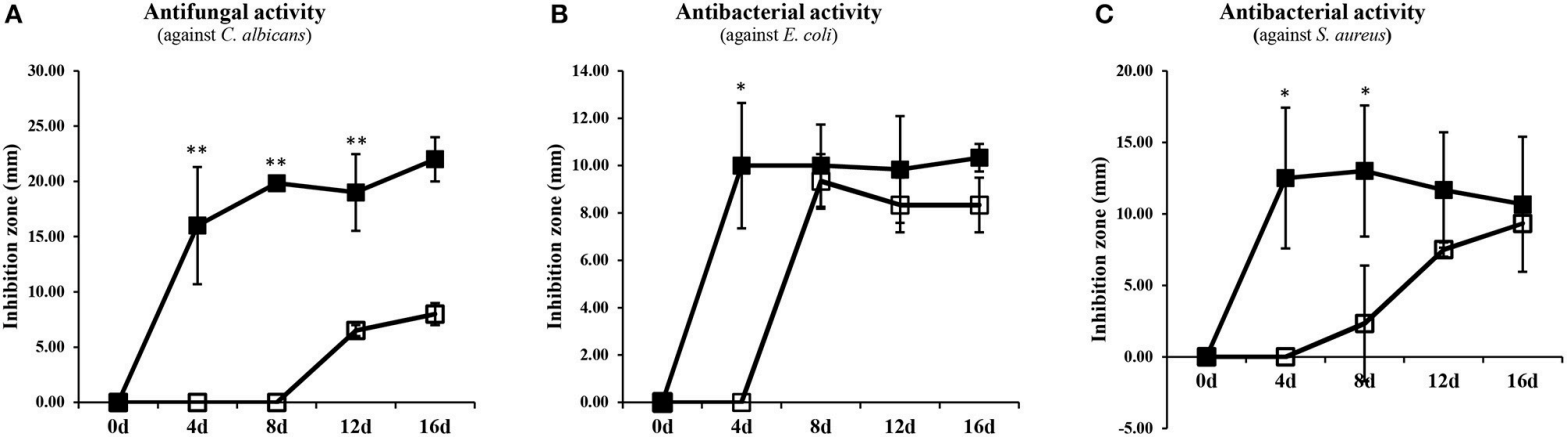
Antimicrobial activity from SSF and SmF extracts (1 mg/100 μL) against **(A)** *Candida albicans*, **(B)** *Escherichia coli*, and **(C)** *Staphylococcus aureus*. The size of disk was 6 mm. Data are shown as means ± S.D (*n* = 3). Significant differences between the SSF and SmF groups were identified by *t*-test (^*^p < 0.05, ^**^p < 0.01). ■, Solid-state fermentation; □, Submerged fermentation.

**Figure 5:**
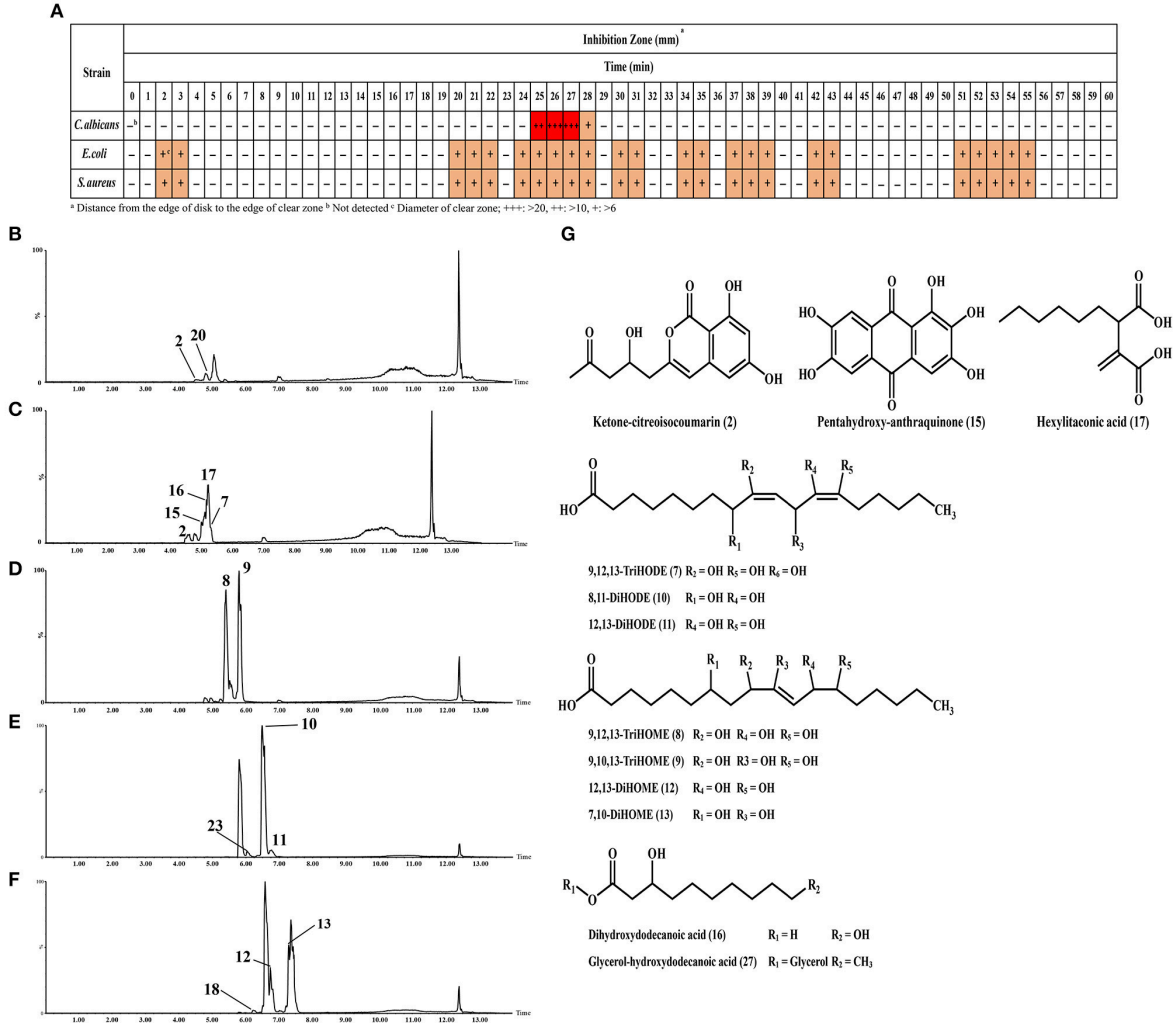
**(A)** Antimicrobial activities (against *C. albicans, E. coli*, and *S. aureus*) for preparative HPLC fractions from the 8 days incubated SSF *A. oryzae* extracts. Chromatograms for **(B)** 22 min, **(C)** 25 min, **(D)** 27 min, **(E)** 31 min, and **(F)** 38 min indicate the bioactive metabolites of each prep fraction. **(G)** Structure of identified metabolites.

The potential antimicrobial activities of SSF and SmF extracts from *A. oryzae* were tested against *C. albicans, S. aureus*, and *E. coli* (Figure 4 and Supplementary Figure 4). Intriguingly, an increasing trend of antimicrobial activities was recorded for the metabolites extracted from both the fermentation states. However, the SSF extracts exhibited the relatively higher antimicrobial activity than those of SmF. Based on the time correlated antimicrobial activities, only 8-day incubated *A. oryzae* SSF extract was further subjected to prep-HPLC analysis and metabolite fractionation (Figure 5). In particular, the fractions collected between 2–3, 20–22, 24–28, 30–31, 34–35, 37–39, 42–43, 51–55 (min) showed notable growth inhibitory activities against *S. aureus* and *E. coli*, whereas fractions between 25–28 (min) selectively inhibited *C. albicans* growth (Figure 5A and Supplementary Figure 5). In particular, we observed that the 25–27 (min) fractions exhibited the highest growth inhibition effects against *C. albicans*. These fractions were further analyzed by UHPLC-LTQ-IT-MS/MS and UPLC-Q-TOF-MS to identify the antimicrobial metabolites. As a result, seven unsaturated fatty acids (7–13), two saturated fatty acids (16, 17), ketone-citreoisocoumarin (2), pentahydroxy-anthraquinone (15), and hexylitaconic acid (18) were variously identified using standard compounds, molecular weight, elemental composition, MS^n^ fragmentation, and UV λ_max_ (nm) data from the collected prep-HPLC fractions (Figures 5B,C and Supplementary Figure 8).

## Discussion

In this study, we unraveled a time correlated metabolomic disparity for secondary metabolites production when *A. oryzae* was cultivated under different culture conditions i.e., SSF and SmF. Moreover, we selected MEA and MEB media to determine the metabolomic differences between SSF and SmF. Both of the medium variants are commonly used for cultivation of filamentous fungi including *Aspergillus* species providing the fermentation environ rich in nutrients supporting their natural growth and sporulation. In general, the SmF is performed using aqueous nutrients, hence presumably suitable for prokaryotes cultivation with planktonic growth (Subramaniyam and Vimala, 2012). The secondary metabolites have been predominantly produced using SmF, even though the corresponding productivity was lesser compared to SSF (Subramaniyam and Vimala, 2012). The SmF has certain associated advantages *viz*., efficient downstream processing, relatively tractable scale-up systems, and easy to control process parameters (pH and aeration), in comparison to SSF (Robinson et al., 2001). However, the usage of SSF for the production of secondary metabolites might not be underestimated. The solid state (or substrate) fermentation is typified by a surface cultivation of cultures on nutrient rich solid support. The SSF is generally employed in the recycling or biovalorization of nutrient-rich solid waste substances (Robinson et al., 2001). In general, the SSF is suitable for filamentous fungi or any microorganism that preferably grows under aerobic conditions with low water activity (Subramaniyam and Vimala, 2012). Previously, the comparative studies on enzyme production, including glucoamylase, protease, and cellulolytic enzymes in *Aspergillus* species under SSF and SmF conditions have been extensively studied (Hansen et al., 2015). Moreover, the studies on primary metabolites, such as organic acids (i.e., succinic acid) and energy sources that directly connect with both cell growth and productivity of secondary metabolites have been investigated (Du et al., 2008). However, a limited number of studies have demonstrated the difference between the nature and the relative abundance of secondary metabolites produced during SSF and SmF involving *A. oryzae*.

### Disparity of secondary metabolite profiles between SSF and SmF

The comparative metabolomic analysis of SSF and SmF extracts of *A. oryzae* demonstrated a marked distinction between secondary metabolite profiles (Figure 1). Overall, twenty-six identified metabolites were classified into three classes of compounds that were observed discriminant between the two fermentation conditions (Table 1). Moreover, all of metabolites were tentatively identified except for metabolite with standard compound such as 12,13-DiHOME (12) and 9 non-identifications. In agreement with our results, the coumarins (1–2), terpenoids (3–6), and unsaturated fatty acids (7–13) have been previously reported from *Aspergillus* species (Schmidt-Dannert, 2015; Costa et al., 2016; Fischer and Keller, 2016). We also examined other secondary metabolites, including diketopiperazines i.e., dihydroxy-methoxyphenylalanine (14), pentahydroxy-anthraquinone (15), dihydroxydodecanoic acid (16), and hexylitaconic acid (18) in *A. oryzae*, reported in different *Aspergillus* spp. (Varoglu and Crews, 2000; Lin et al., 2008; Senthilkumar et al., 2014; Fouillaud et al., 2016).

The production of secondary metabolites in filamentous fungi varies remarkably depending on their different culture conditions. Herein, we observed a marked disparity in the nature and abundance of significantly discriminant secondary metabolites produced under SSF and SmF conditions for *A. oryzae*. In particular, the relative abundance of coumarins (1–2), unsaturated fatty acids (7–13), diketopiperazine derivatives (14), pentahydroxy-anthraquinone (15), dihydroxydodecanoic acid (16), and hexylitaconic acid (18), were observed selectively higher in SSF. Among the different metabolite classes, coumarins broadly are represented by benzopyrone compounds with fused benzene and alpha-pyrone rings. Approximately 1300 coumarins have been identified as secondary metabolites from plants, bacteria, and fungi (Costa et al., 2016). Functionally, coumarins are attractive candidates for combinatorial biosynthesis owing to their established biological activities *viz*., antioxidant, antibacterial, antifungal, and antiulcer potentials (Costa et al., 2016). Previously, six *Aspergillus* species were evaluated under different culture conditions i.e., resting (similar to SSF) and submerged culture (relatively disturbed, similar to SmF) states, with the prior one showing higher coumarin bioconversion rates (Costa et al., 2016). Hence, we suggest that the resting state of mycelia in SSF might have resulted in an enhanced biosynthesis of coumarins.

On the other hand, terpenoids were relatively higher in SmF extracts than those in SSF extracts. The terpenoid compounds *viz*., sesquiterpenoids, diterpenoids, triterpenoids, and meroterpenoids exhibit a range of bioactivities including antibacterial, antifungal, antitumor, and anticancer (Schmidt-Dannert, 2015). In this study, we distinctively identified asperaculin A (sesquiterpenoid) and austalide F and H (meroterpenoids) in *A. oryzae*. All of these secondary metabolites were previously reported from marine-derived *Aspergillus* species (Ingavat et al., 2011; Schmidt-Dannert, 2015; Peng et al., 2016). Many reports have been published on the optimization of submerged fermentation to produce bioactive terpenoids (Xiao and Zhong, 2016). In particular, it was reported that the titer of ganoderic acid (GA) and oxygenated lanostane-type triterpenoid was affected by pH shifts and dissolved oxygen tension (DOT) levels, illustrating the effects of cultivation parameters and physiological states on metabolite production (Xiao and Zhong, 2016). Typically, at the beginning of submerged fermentation, the amount of oxygen is high, but as the fermentation progresses, the pH and oxygen level decrease during SmF, and hence the terpenoid contents are expected increase during later stage of SmF (Tang et al., 2009; Vendruscolo et al., 2012; Arumugam et al., 2014).

The unsaturated fatty acids play important roles in prokaryotic and eukaryotic organism, such as cell proliferation, apoptosis, tissue repair, inflammation, and immune cell behavior (Tsitsigiannis and Keller, 2007). The oxygen containing unsaturated fatty acids, trivially the oxylipins, mediate crucial biological activities, including both the intra- and inter-cellular signaling in plants, vertebrates, invertebrates, and fungi (Tsitsigiannis and Keller, 2007). Functionally, the oxylipins are known to exhibit many pharmacological activities *viz*., antimicrobial and anti-inflammatory (Tsitsigiannis and Keller, 2007). In fungi, the oxylipins regulate cell growth, modulation of spore shape, and germination rate, as well as maintain the ratio of sexual to asexual spore development (Fischer and Keller, 2016). Although the comparative studies profiling the oxylipin class metabolites between SSF and SmF are limited, the levels of the index of unsaturated fatty acids (IUFA, a fatty acid precursor of oxylipins) were reportedly higher in SSF than SmF, and hence the concentration of oxylipin ought to be relatively higher in SSF (Zhang et al., 2015).

Moreover, several miscellaneous discriminant metabolites *viz*., diketopiperazine derivatives (14), pentahydroxy-anthraquinone (15), dihydroxydodecanoic acid (16), and hexylitaconic acid (18) were observed relatively abundant in SSF. Although, the studies describing the effects of culture conditions on these discriminant metabolites are limited, the higher production of bio-pigments was reported in SSF compared to SmF, potentially justifying the relatively higher abundance of pentahydroxy-anthraquinone (a natural pigment) under SSF in the present study (Fouillaud et al., 2016; Sehrawat et al., 2017). In addition, the rest of the three distinctive metabolites might also have conferred some adaptive and cryptic function in cultivated molds. Most of these metabolites were previously reported from *Aspergillus* species, reportedly exhibiting a wide range of bioactivities (Varoglu and Crews, 2000; Lin et al., 2008; Senthilkumar et al., 2014). However, the adaptive functions and physiological alteration under different culture conditions need to be studied further in terms of individual functions of metabolite classes.

### Antimicrobial compounds and their efficacies

We examined overall 12 metabolites, identified from the active prep-HPLC fractions of SSF extracts, positively exhibiting antimicrobial activities. Positively exhibiting antimicrobial active metabolites were also identified tentatively except for metabolite with standard compound. Owing to the relatively higher and earlier display of antimicrobial activities (Figure 4), we selectively performed prep-HPLC fractionation for 8-day incubated SSF extracts toward the separation of bioactive compounds. Recently, the coumarins related to ketone-citreoisocoumarin (2) were isolated from various organisms, and their antimicrobial activities were established against *E. coli* and *C. albicans* (Costa et al., 2016). The parent “coumarin” structure has not only the ability of non-covalent interactions (hydrophobic and electrostatic interactions, hydrogen bonds, and van der Waals force, among others) with many active sites, but also pharmacological activities, such as antimicrobial and antioxidant, among others (Barot et al., 2015). Earlier, it was reported that the hydroxylated coumarins at the C-6, C-7, and C-8 position inhibit bacterial growth through damaging the cell membrane (Yang et al., 2016a).

In agreement with our results, the oxygenated fatty acids (oxylipins) were previously reported to exert antibacterial and antifungal effects (Tsitsigiannis and Keller, 2007). Recently, Ma et al. (2016) have established the antifungal mechanisms for synthetic compounds mimicking the natural oxylipins, proposed that the linear aliphatic compound with more -OH groups efficiently interact with fungal cytoderm showing higher inhibitory activity. Among the saturated fatty acids, dihydroxydodecanoic acid (16) and glycerol-hydroxydodecanoic acid (27) were previously reported to exhibit antibacterial activity (Smith et al., 2008). The saturated fatty acid with a C-9 straight-chain, dodecanoic acid (lauric acid), had associated antibacterial activity, which specifically disrupts bacterial growth. Moreover, the esterification of dodecanoic acid with glyceryl to form glycerides also demonstrated antimicrobial activities (Smith et al., 2008).

Among the polyketides, anthraquinone and hydroxyanthraquinone, specifically exhibited high antimicrobial activities. As previously reported, anthraquinone derivatives related to pentahydroxy-anthraquinone (15) inhibited the growth of *S. aureus* and *E. coli* (Fouillaud et al., 2016). Anthraquinone belongs to a class in the quinone family, with its structure containing three benzene rings coupled with two ketone groups attached to the central ring. The higher degree of oxidative stress induced by these compounds might be linked to the reported antibacterial and antifungal activities (Fouillaud et al., 2016). Further, hexylitaconic acid (18) from an endophytic fungus, *Eupenicillium* sp. LG41, exhibited noticeable efficacy against *Acinetobacter* sp. BD4, *S. aureus*, and *E. coli* (Li et al., 2014). Additionally, the N.I. 2 (20) might be inhibited the growth of *S. aureus* and *E. coli*. Based on the relatively higher abundance of bioactive metabolite production in SSF and their established antimicrobial potentials, we can speculate why *Aspergillus* emerges as the dominant genus during the course of fermentation and exhibiting a similar effect as a biopreservative in fermented food. We putatively identified the specific bioactive metabolites, which could confer a competitive edge to *A. oryzae*, during colonization of fermentative substrates. However, the present hypothesis needs to be firmly established through more comprehensive metabolite profiling and correlation with their functional and antimicrobial activities in additional studies using diverse strains relevant to the commercial fermentation practices.

In conclusion, we evaluated the non-targeted metabolomic profiles using LC-ESI-MS/MS and antimicrobial activities of *A. oryzae* 12698 under different fermentation states (SSF and SmF). This approach demonstrated that the production of secondary metabolites in *A. oryzae* was significantly affected by different cultivation conditions with SSF observed to be noteworthy in relation to antimicrobial metabolite production. In addition, specific bioactive metabolites were putatively identified that could confer a competitive edge to *A. oryzae* during colonization on fermentative substrates. This study provides a metabolomic framework underpinning the growth and bioactive compound production in *A. oryzae* in different culture conditions. Furthermore, the comprehensive profiling of the secondary metabolites might illuminate the range of secreted metabolic classes helpful in delineating the optimal fermentative conditions and associated implications of secreted end products, either beneficial or detrimental, especially in relation to fermented foods and beverages involving *Koji* mold.

## Author contributions

CL designed this research. SS performed the experiments and data analysis. SS, SL, DS, N-RL, and D-YL conducted the data interpretation. The samples of *Aspergillus oryzae* KCCM 12698 were procured from the Korean Culture Center of Microorganism. The remainder of microorganisms, of *Candida albicans* KACC 30062, *Staphylococcus aureus* KACC 1621, and *Escherichia coli* KCTC 1682, were provided by the Korean Agricultural Culture Collection and the Korean Collection for Type Cultures. SS wrote the paper. All authors approved the final manuscript.

### Conflict of interest statement

The authors declare that the research was conducted in the absence of any commercial or financial relationships that could be construed as a potential conflict of interest.

## Abbreviations

SSF: Solid state fermentation
SmF: Submerged fermentation
UHPLC-LTQ-IT-MS/MS: Ultra high-performance liquid chromatography-linear trap quadrupole-ion trap-tandem mass spectrometry
UPLC-Q-TOF-MS: Ultraperformance liquid chromatography-quadrupole-time of flight-mass spectrometry
PCA: Principal component analysis
PLS-DA: Partial least squares projection to latent structures discriminant analysis
