# Supplementary material for: Comprehensive Secondary Metabolite Profiling Toward Delineating the Solid and Submerged-State Fermentation of Aspergillus oryzae KCCM 12698
Source: Front Microbiol. 2018 May 25;9:1076. doi: 10.3389/fmicb.2018.01076 (PMC5981208; doi:10.3389/fmicb.2018.01076)
Supplement: Supplementary file 1 [file Presentation_1.pdf]

Supplemental Material

Comprehensive Secondary Metabolite Profiling Toward Delineating the Solid and Submerged-state Fermentation of *Aspergillus oryzae* KCCM 12698

Su Young Son [1], Sunmin Lee [1], Digar Singh [1], Na-Rae Lee [2], Dong-Yup Lee [2], and Choong Hwan Lee [1*]

[1] Department of Bioscience and Biotechnology, Konkuk University, Seoul, South Korea
[2] NUS Synthetic Biology for Clinical and Technological Innovation (SynCTI), Life Sciences Institute, National University of Singapore, 28 Medical Drive, Singapore 117456, Singapore

[*] Correspondence:
Tel.: +82-2-2049-6177; Fax: +82-2-455-4291; E-mail address: chlee123@konkuk.ac.kr

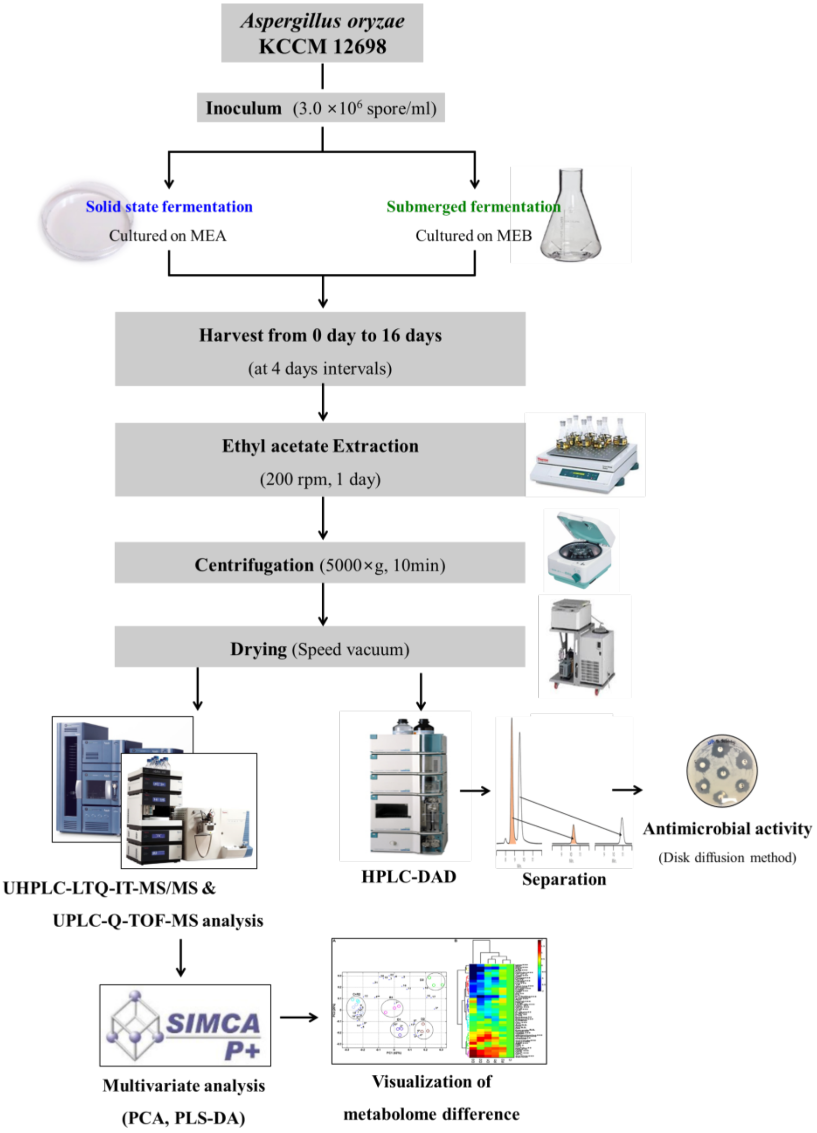

Supplementary Figure 1. Schematic representation of experimental procedure.

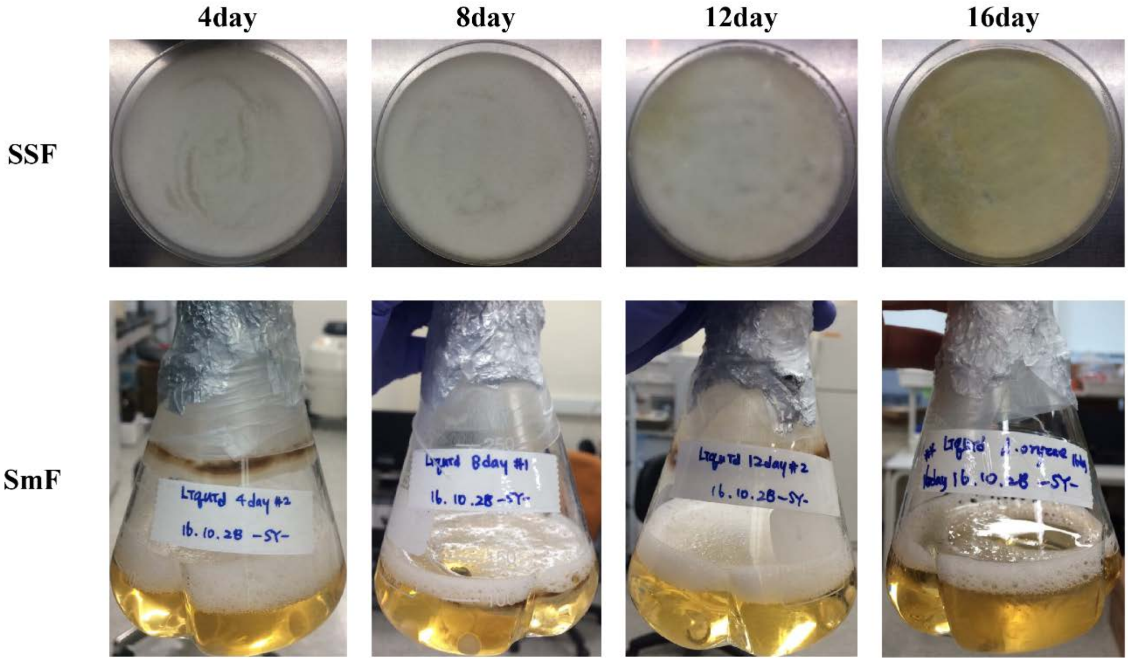

Supplementary Figure 2. Time-resolved (4, 8, 12, 16 day) growth of *A. oryzae* KCCM 12698 on Malt Extract Agar (MEA) for SSF and Malt Extract Broth (MEB) for SmF.

SSF, solid state fermentation; SmF, Submerged fermentation

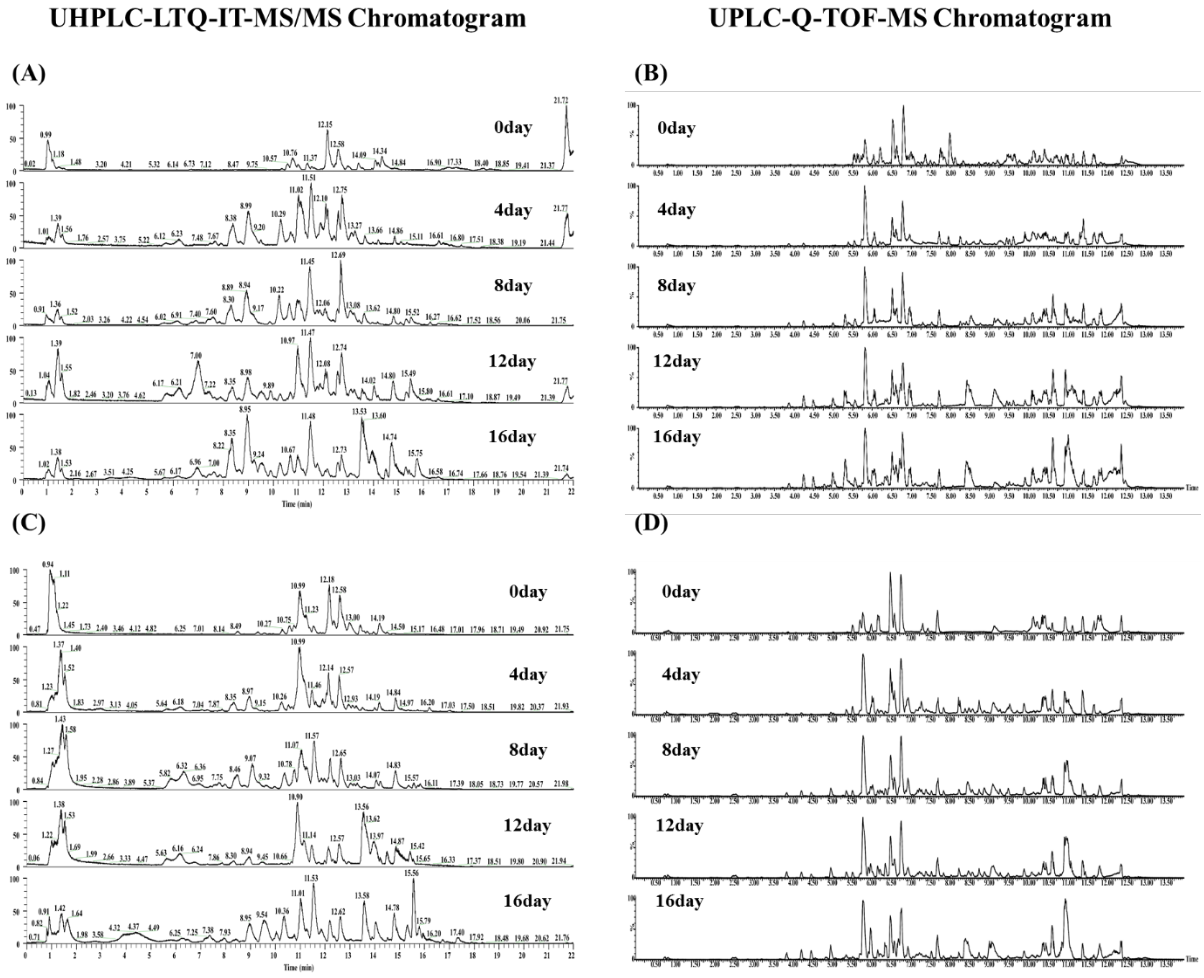

Supplementary Figure 3. The SSF Chromatogram of *A. oryzae* KCCM 12698 extracts analyzed by (A) UHPLC-LTQ-IT-MS/MS and (B) UPLC-Q-TOF-MS, and SmF chromatogram of *A. oryzae* KCCM 12698 extracts analyzed by (C) UHPLC-LTQ-IT-MS/MS and (D) UPLC-Q-TOF-MS. All chromatogram is negative ion mode.

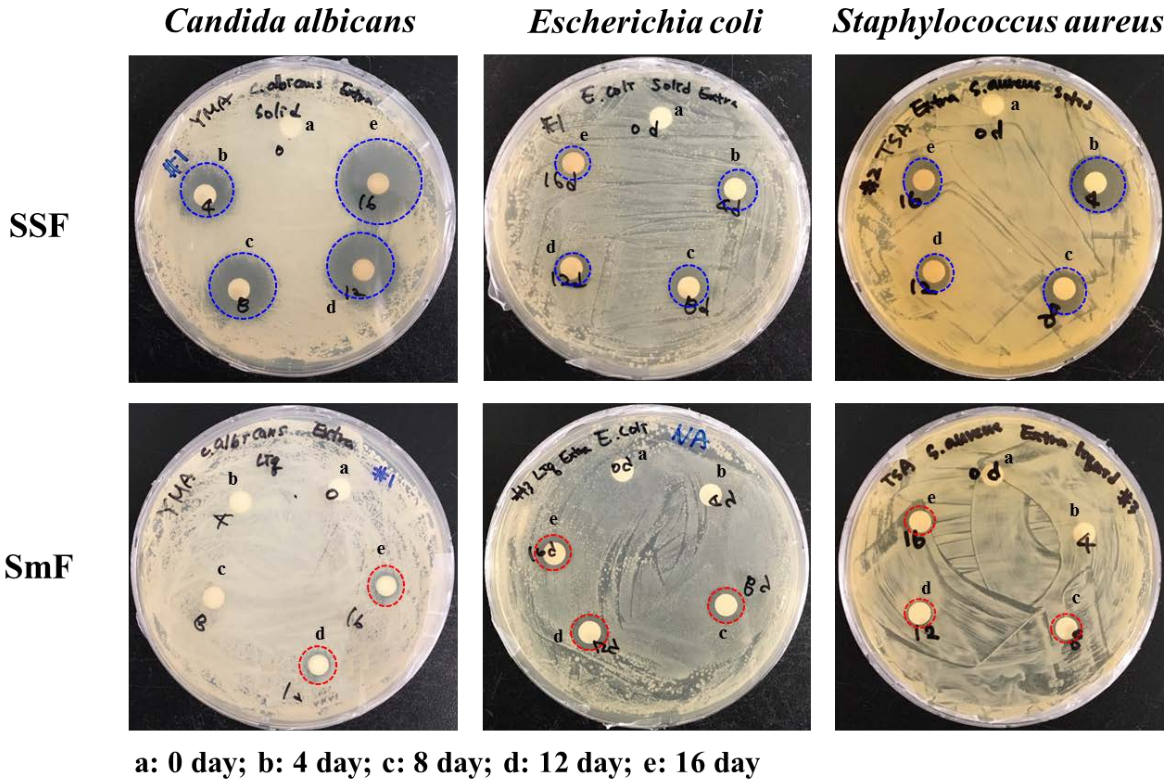

Supplementary Figure 4. Representative photographs showing the zone of inhibition for the metabolites extracted from the time-resolved (0, 4, 8, 12, 16 day) SSF and SmF samples.

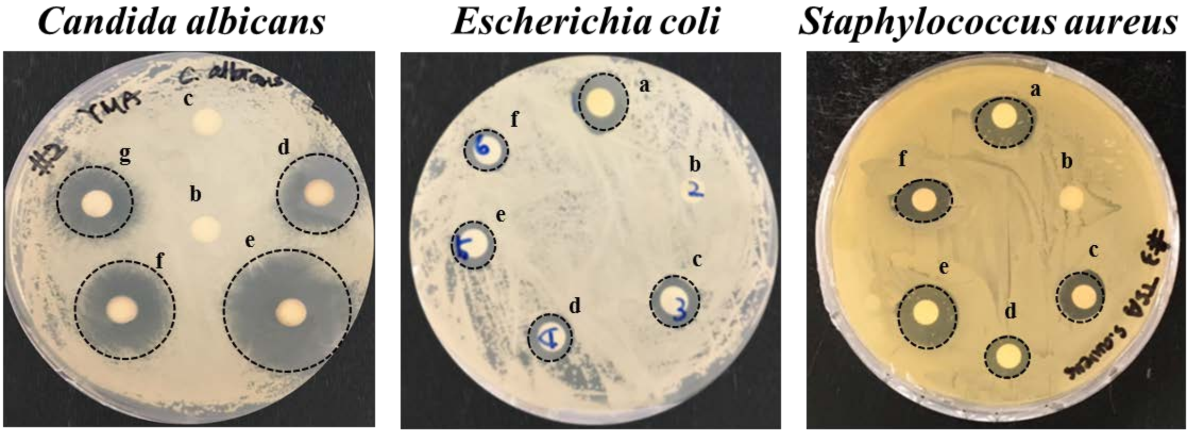

a: 22min; b: 23 min; c: 24 min; d: 25 min; e: 26 min; f: 27 min; g: 28 min

Supplementary Figure 5. Representative photographs showing the zone of inhibition of active prep-HPLC fraction (22-28min).

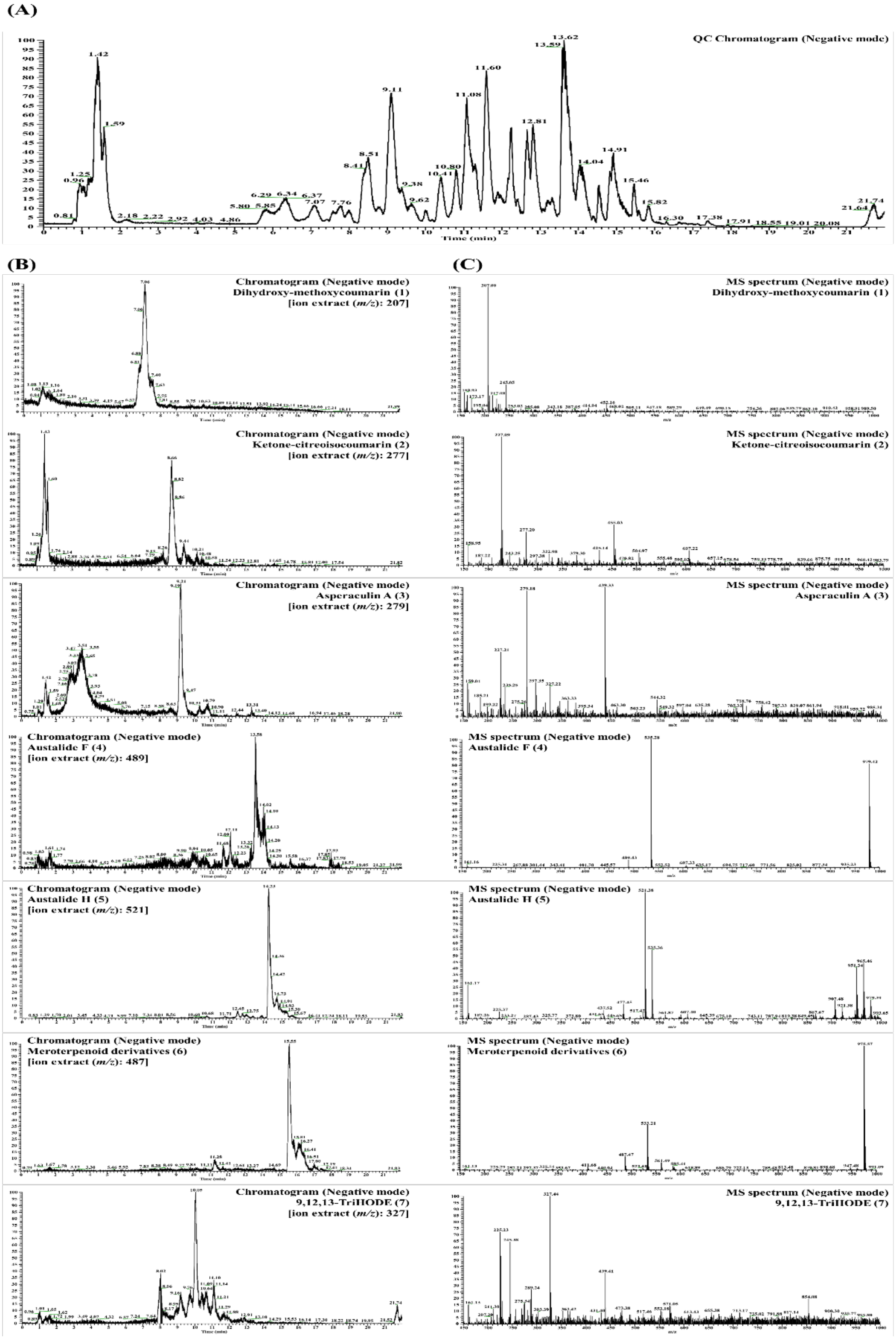

Supplementary Figure 6. Continued

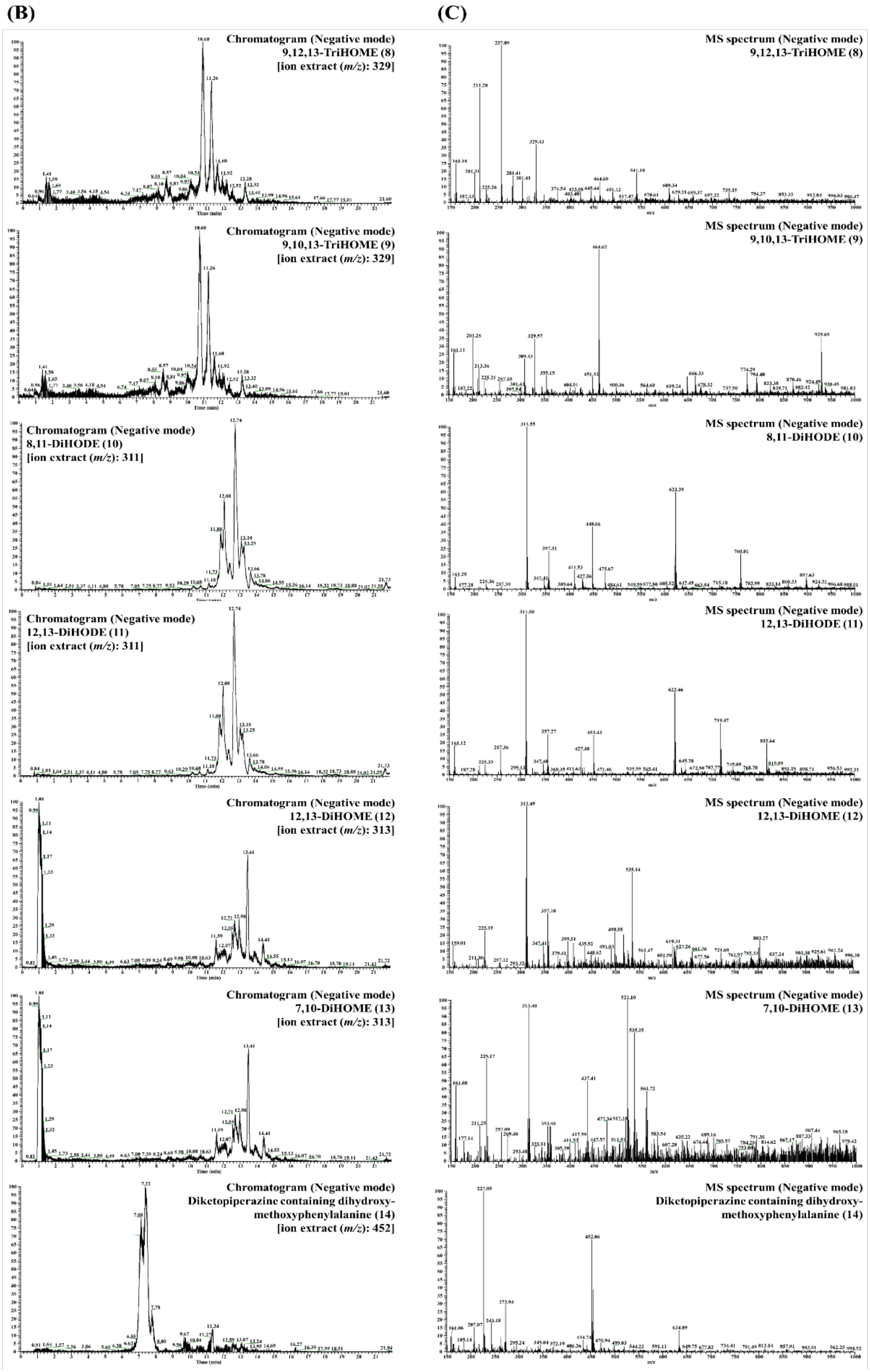

Supplementary Figure 6. Continued

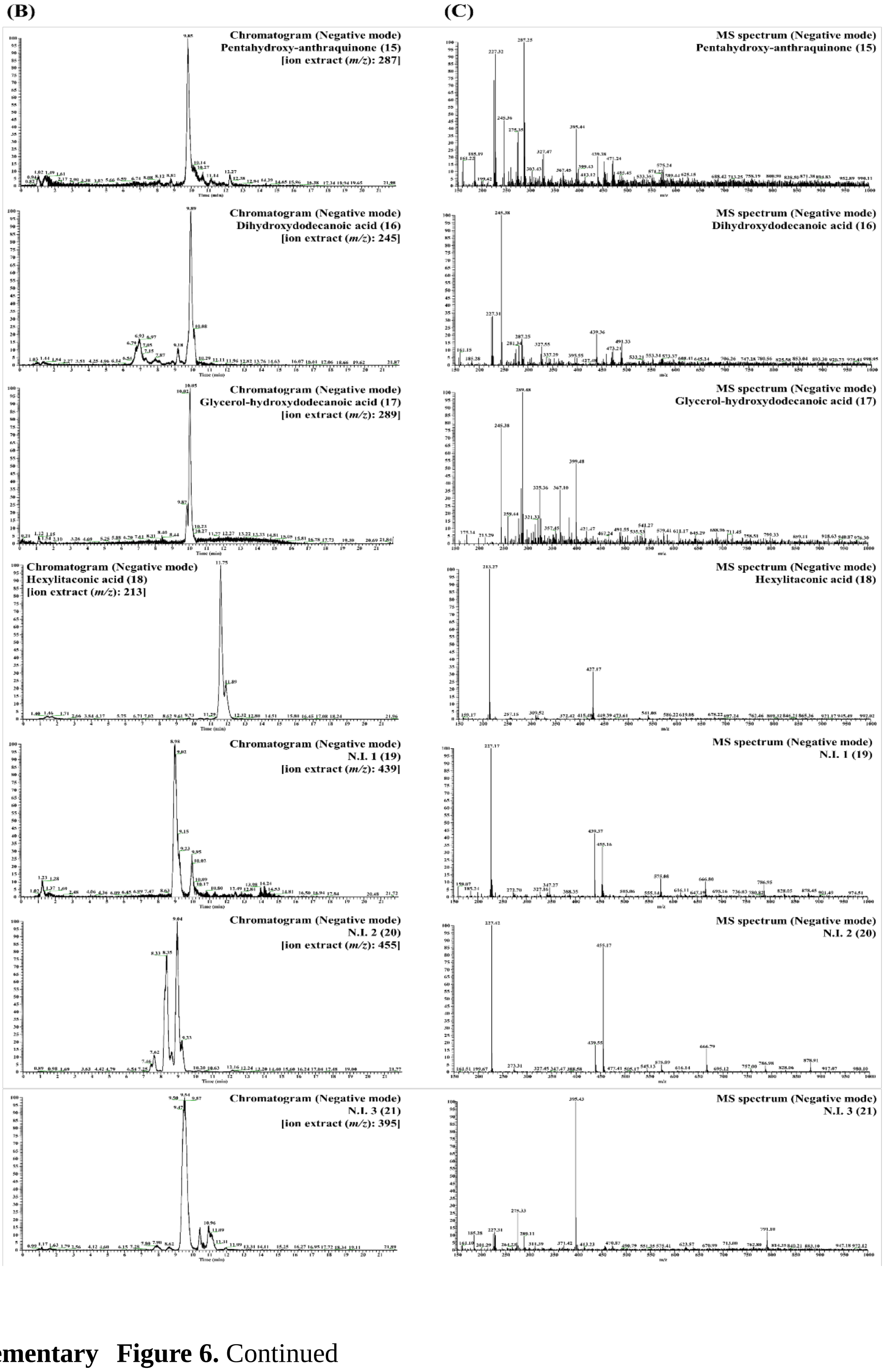

Supplementary Figure 6. Continued

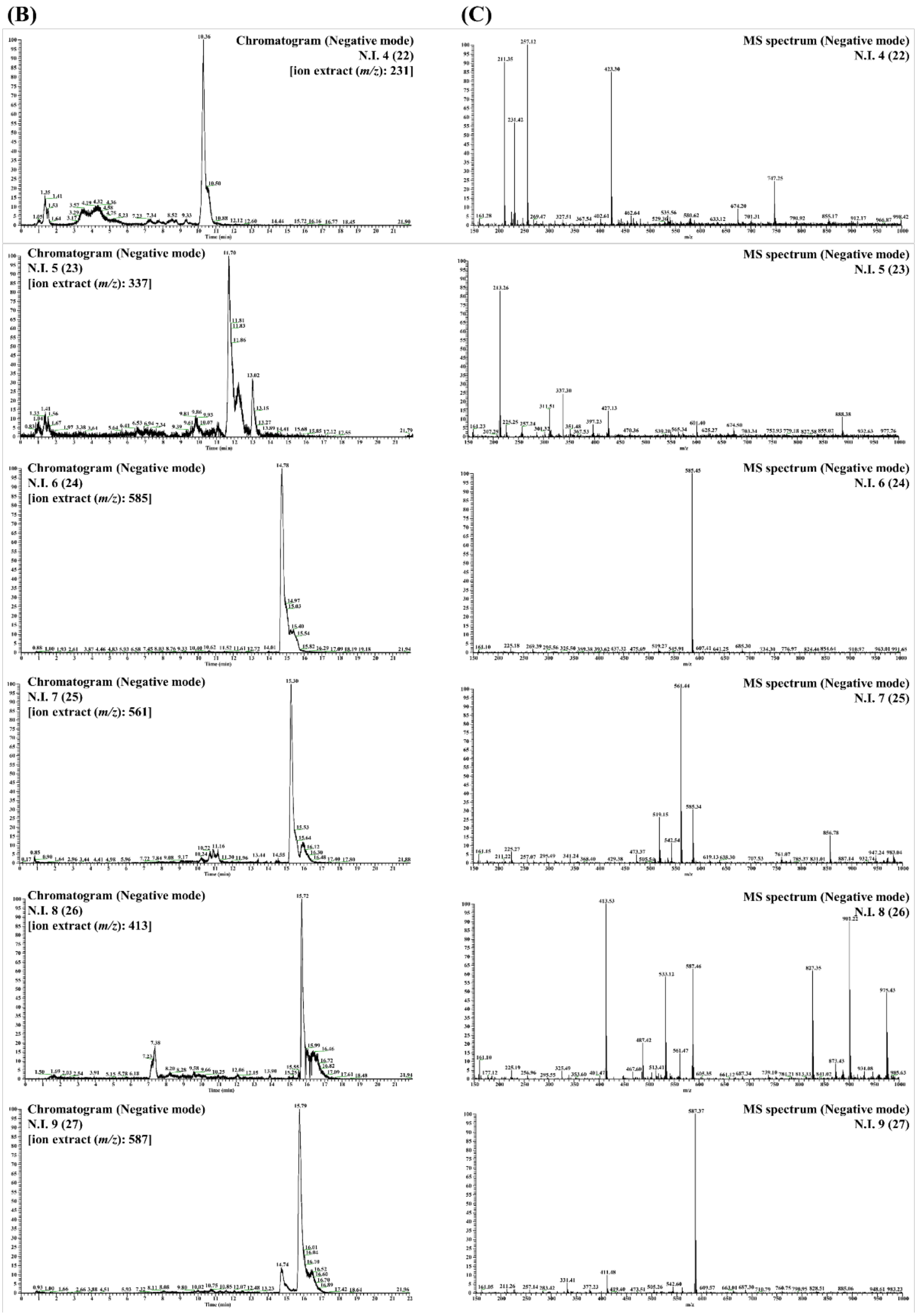

Supplementary Figure 6. The chromatogram of quality control (QC) sample (A). Ion extracted chromatogram of tentatively identified metabolites (B). MS spectrum of tentative identified metabolites (C). All of chromatograms and spectrum were negative ion mode.

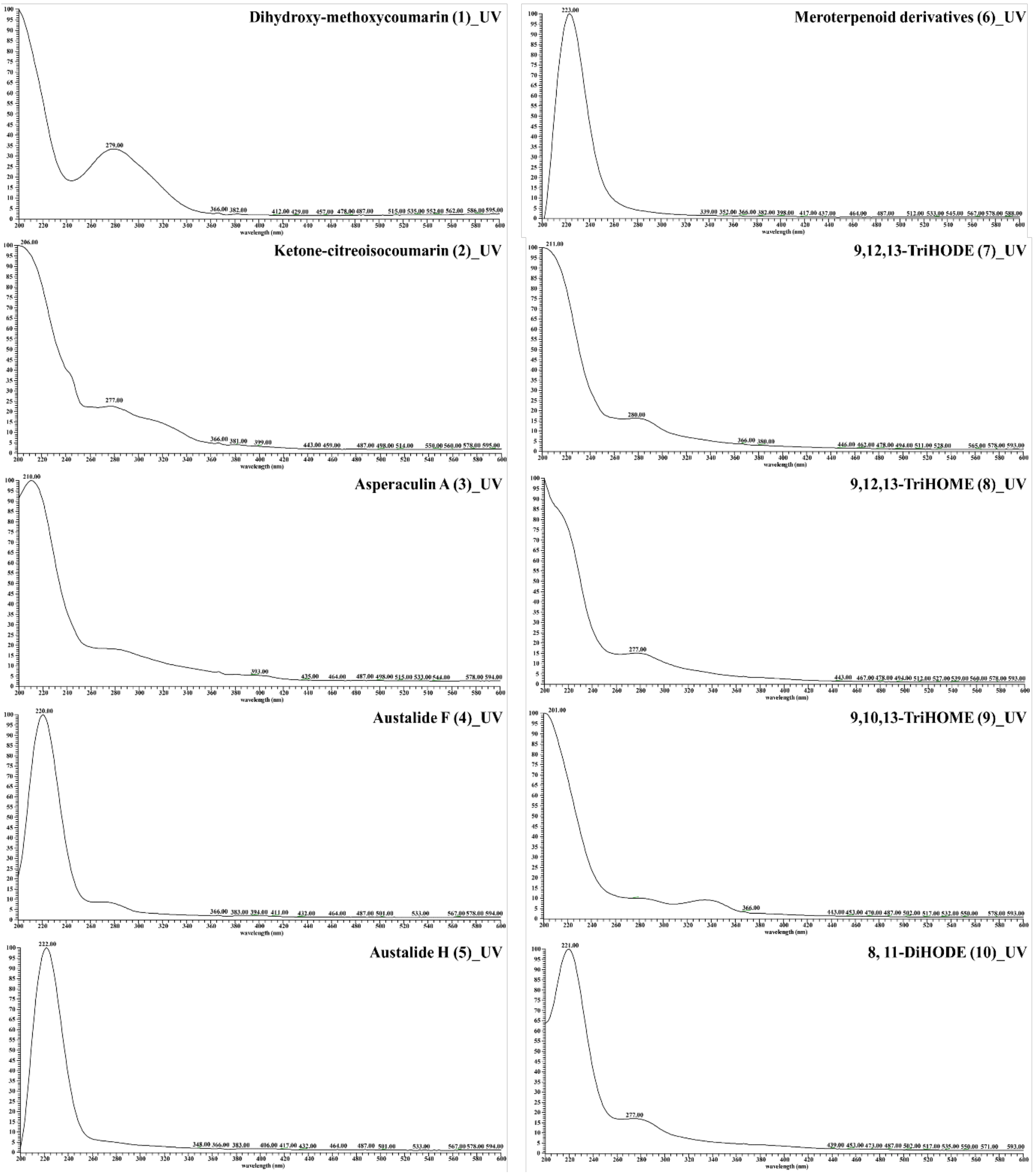

Supplementary Figure 7. Continued.

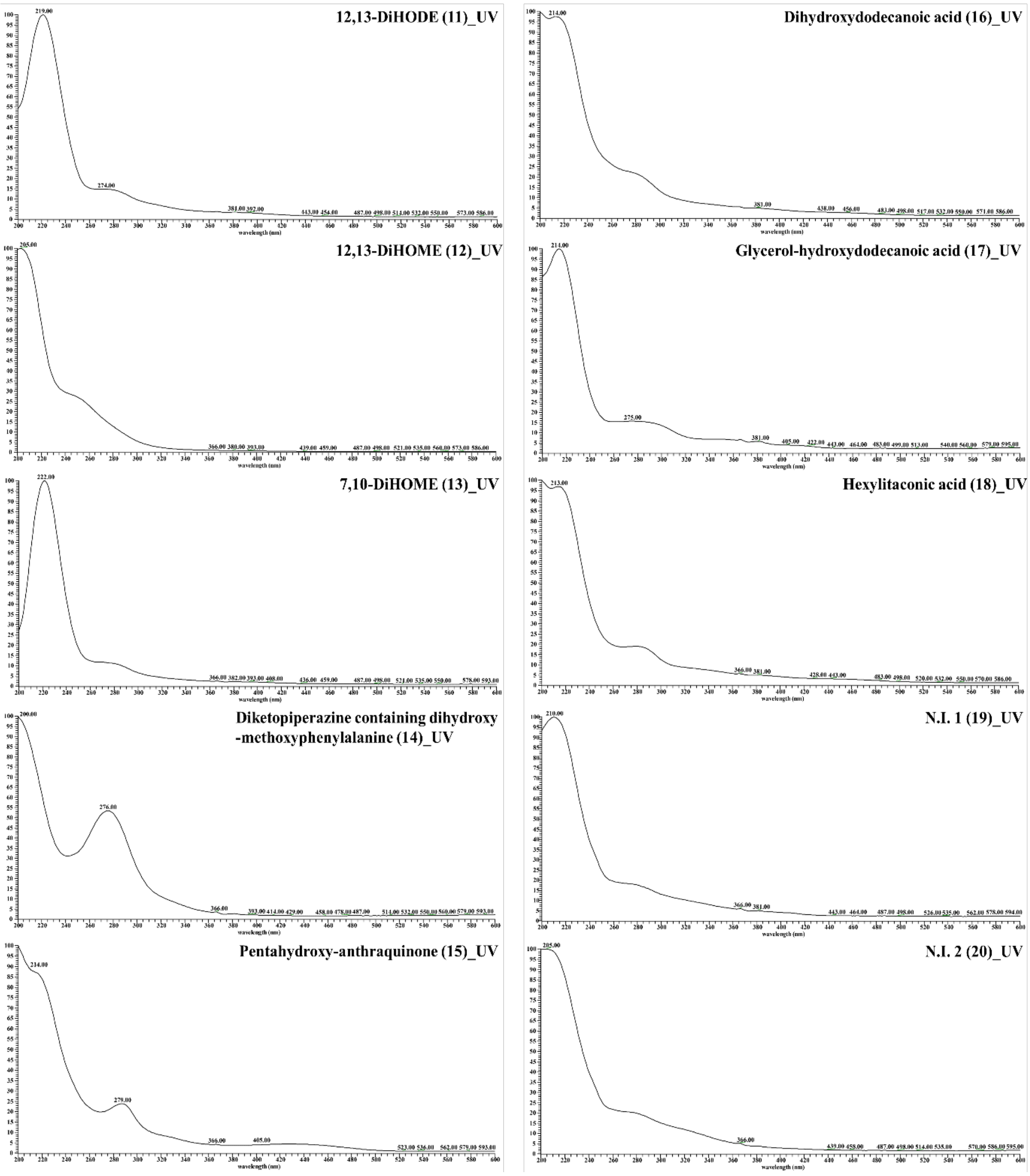

Supplementary Figure 7. Continued.

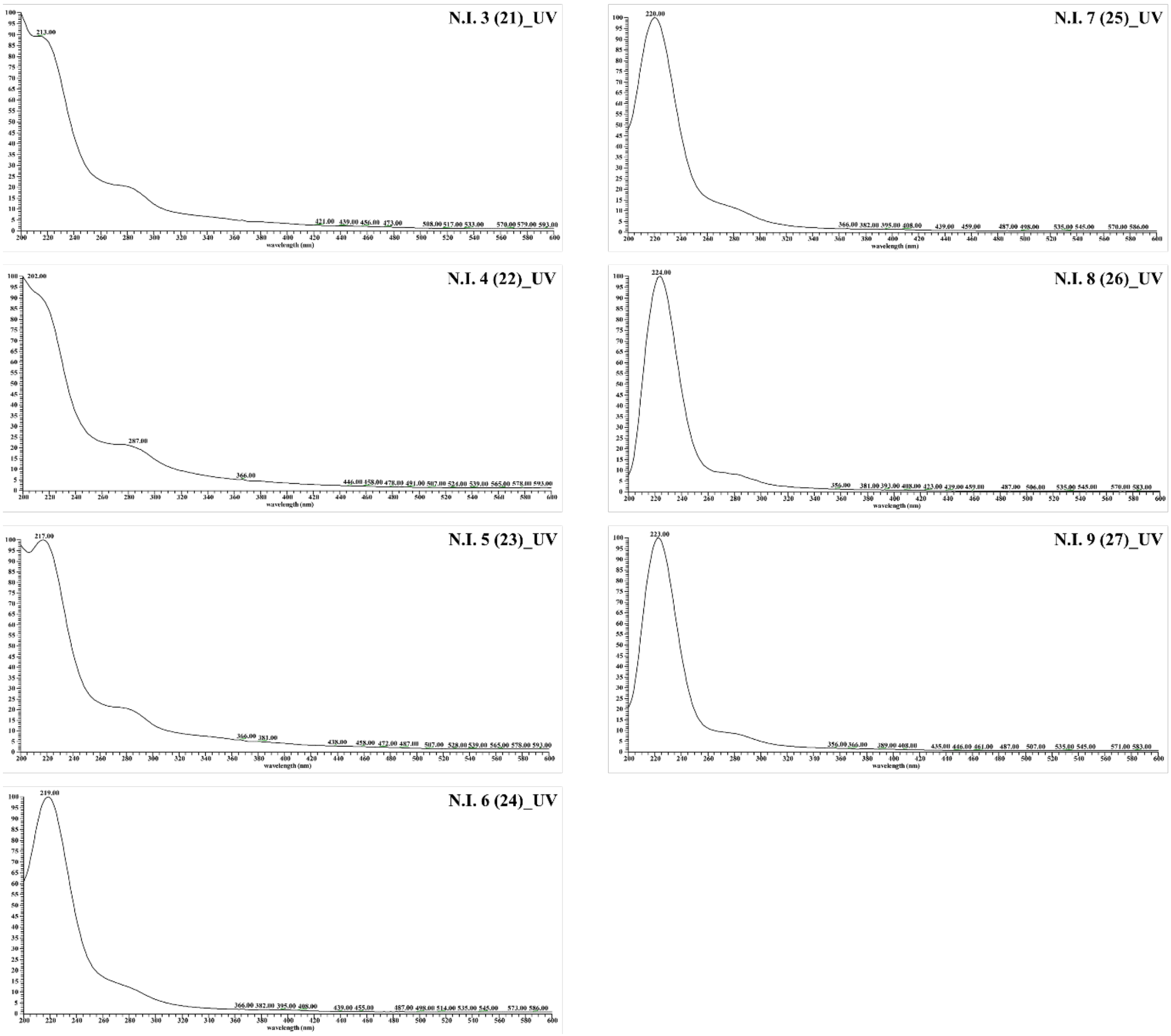

Supplementary Figure 7. UV Spectrum of tentatively identified metabolites.

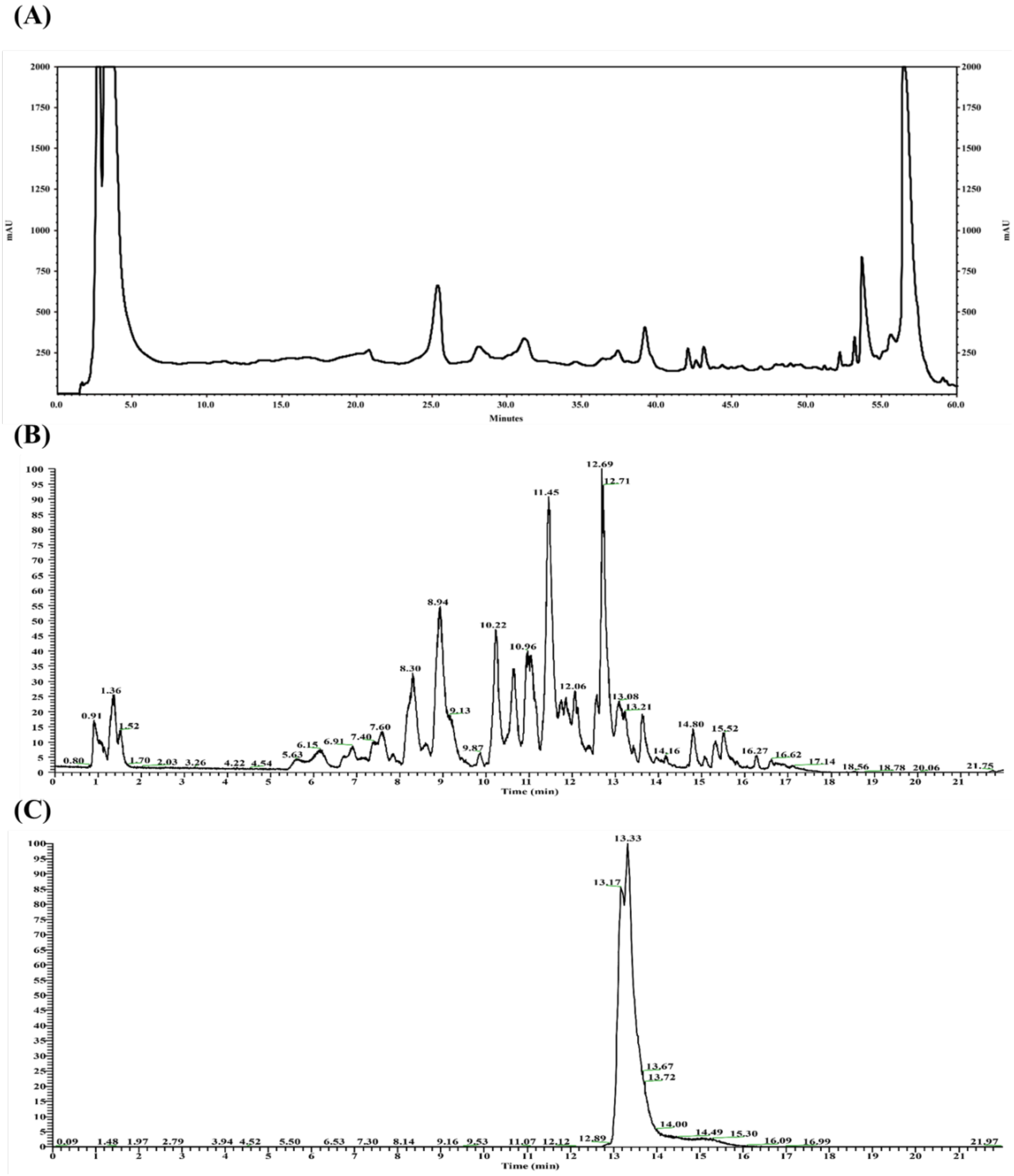

Supplementary Figure 8. (A) The chromatogram of *A. oryzae* KCCM 12698 of SSF 8 day crude extracts analyzed by HPLC-DAD at λ_{max} 220, (B) The chromatogram of *A. oryzae* KCCM 12698 of SSF 8 day crude extracts analyzed by UHPLC-LTQ-IT-MS/MS in negative mode, (C) The chromatogram of STD compound (12,13-DiHOME (12)) analyzed by UHPLC-LTQ-IT-MS/MS in negative mode.
